# Clinicopathological Characteristics and Survival Outcomes in Patients With Gastric Adenocarcinoma: A 16-Year Experience From a Single Tertiary Center in Tunisia

**DOI:** 10.7759/cureus.106194

**Published:** 2026-03-31

**Authors:** Amal Bouazzi, Sami Lagha, Marwa Azri, Mahdi Beltaifa, Waad Farhat, Fathia Harrabi, Abdelkader Mizouni, Nabiha Missaoui, Mohamed Ben Mabrouk

**Affiliations:** 1 Department of General Surgery, Sahloul University Hospital, Medicine Faculty of Sousse, University of Sousse, Sousse, TUN; 2 Research Laboratory LR21ES03, Oncogenesis and Tumor Progression, Medicine Faculty of Sousse, University of Sousse, Sousse, TUN

**Keywords:** gastrectomy, gastric adenocarcinoma, overall survival, prognostic factors, recurrence patterns, tunisia

## Abstract

Introduction: Gastric cancer remains a leading cause of cancer-related mortality worldwide, with marked geographic disparities in presentation and outcomes. Surgical resection remains the cornerstone of curative treatment; however, survival and recurrence patterns vary according to clinicopathological features.

Objective: This study aimed to evaluate overall survival and recurrence outcomes after gastrectomy for patients with gastric adenocarcinoma and to identify independent prognostic factors in a North African tertiary-care setting.

Material and methods: We conducted a retrospective cohort study spanning 16 years, including 192 patients who underwent curative-intent or palliative gastrectomy for histologically confirmed gastric adenocarcinoma at a single tertiary center in Tunisia. Overall survival, locoregional recurrence, and distant metastasis were analyzed via the Kaplan-Meier method and Cox proportional hazards regression.

Results: The overall survival rates were 57.5% at one year, 22% at five years, and 14% at 10 years. On multivariate analysis, residual tumor, larger tumor size, and advanced pathological stage were independently associated with poorer survival. Locoregional recurrence occurred in 21.7% of patients and was independently associated with subtotal gastrectomy (hazard ratio (HR) = 2.81). Distant recurrence was observed in 41.4% of the patients and was independently associated with serosal invasion (HR = 2.88; p = 0.023) and an infiltrative tumor growth pattern (HR = 3.49; p = 0.002).

Conclusion: These findings underscore the need for optimized surgical strategies and tailored multimodal treatment approaches, particularly in regions where advanced-stage presentation remains common.

## Introduction

Gastric cancer (GC) remains a major global health burden, with marked disparities between developed and developing countries in terms of incidence, stage at diagnosis, and survival outcomes [1,2]. According to GLOBOCAN 2020, it is the fifth most frequently diagnosed cancer and the third leading cause of cancer-related mortality worldwide, accounting for approximately 1.1 million new cases and 770,000 deaths annually [3,4]. Although the incidence is highest in East Asia, global demographic shifts and the persistence of established risk factors are projected to drive a 62% increase in new cases by 2040, particularly in low- and middle-income countries [3]. Despite therapeutic advances, the overall five-year survival rate for patients with GC remains below 30% in most populations, underscoring the need for improved strategies across the continuum of care [5].

The epidemiology of GC is characterized by substantial geographic heterogeneity. Age-standardized incidence rates range from fewer than five per 100,000 in North America to more than 30 per 100,000 in Eastern Asia, the Andean regions, and post-Soviet states [6]. While industrialized countries have experienced a sustained decline in incidence (largely attributed to reduced *Helicobacter pylori *(*H. pylori*) prevalence, improved food preservation, and lifestyle modifications), many developing regions continue to face delayed epidemiological transition, lagging by nearly three decades [7]. The Middle East and North Africa (MENA) region exhibits a distinct pattern, with moderate incidence but disproportionately high mortality, reflected by a mortality-to-incidence ratio exceeding 0.75 [8,9]. This imbalance likely reflects late-stage presentation, limited access to diagnostic endoscopy, and fragmented referral pathways to specialized oncologic care, emphasizing the need for region-specific outcome data.

From a biological and clinical standpoint, GC is a heterogeneous disease. While molecular classifications such as those proposed by The Cancer Genome Atlas have refined our understanding of tumor biology, clinical outcomes in routine practice remain largely driven by anatomical extent, histopathological features, and resection quality [10]. Established etiological factors-including *H. pylori* infection, dietary nitrosamines, gastroesophageal reflux disease, and obesity-show marked regional variation [11]. Among these, *H. pylori* remains the most significant carcinogenic factor; *H. pylori* is implicated in approximately 89% of noncardia GC cases and is classified as a Group I carcinogen [12].

Surgical resection with adequate lymphadenectomy remains the cornerstone of curative treatment for localized GC, with D2 lymphadenectomy now considered standard practice on the basis of evidence from major trials such as CLASSIC (Capecitabine and Oxaliplatin Adjuvant Study in Stomach Cancer) and ARTIST (Adjuvant Chemoradiation Therapy in Stomach Cancer) [13,14]. Nevertheless, disease recurrence occurs in approximately 40-60% of patients even after R0 resection, most commonly as peritoneal or hepatic metastases [15]. Although perioperative chemotherapy regimens, including FLOT (fluorouracil (5-FU), leucovorin (also known as folinic acid), oxaliplatin, and docetaxel), have improved survival in selected patients, outcomes remain highly variable, reflecting tumor biology, stage at diagnosis, and treatment-related factors. Identifying clinicopathological predictors of survival and recurrence, therefore, remains essential, particularly in real-world settings where access to multimodal therapy may be inconsistent [15].

In this context, robust regional data are needed to better characterize oncological outcomes and support evidence-based clinical decision-making in resource-limited settings. Accordingly, this retrospective cohort study aimed to evaluate survival and recurrence outcomes in patients with GC undergoing curative gastrectomy at a single tertiary center in Tunisia. The specific objectives were to (i) assess overall survival following surgery, (ii) identify clinicopathological factors independently associated with overall survival, and (iii) determine predictors of postoperative disease recurrence, including locoregional recurrence and distant metastasis. By addressing these objectives, the study provides region-specific prognostic data that may improve risk stratification and inform postoperative management strategies in similar healthcare settings.

## Materials and methods

Study design and setting

This study was designed as a retrospective cohort study conducted over 16 years, from January 1, 2000, to December 31, 2015, in the General Surgery Department of our University Hospital. Ethical approval was waived by the local ethics committee of Sahloul University Hospital due to the retrospective study design and the exclusive use of anonymized data. The study complied with the Tunisian Law No. 2004-63 of July 27, 2004, on the protection of personal data and with institutional policies governing the use of clinical data for research. All procedures were conducted in accordance with the principles of the Declaration of Helsinki, and patient data were fully anonymized prior to analysis to ensure confidentiality. The study is reported in compliance with the Strengthening the Reporting of Observational Studies in Epidemiology (STROBE) guidelines.

Study population

All consecutive patients with histologically confirmed gastric adenocarcinoma who underwent surgical management with curative or palliative intent during the study period were eligible. Patients with adenocarcinomas involving the esophagogastric junction were excluded to ensure cohort homogeneity, as were patients with incomplete medical records precluding assessment of key clinicopathological or outcome variables. Eligible patients were identified through archived operative records and pathology databases.

Data collection

Clinical data were collected retrospectively via a standardized and predefined data collection form. The variables included demographic characteristics, comorbidities, presenting symptoms, laboratory findings, radiological and endoscopic assessments, surgical procedures, neoadjuvant and adjuvant treatments, intraoperative findings, histopathological characteristics, postoperative morbidity and mortality, and follow-up outcomes.

Follow-up data were obtained from outpatient clinic visits and hospital records. For patients who were lost to scheduled follow-up, survival status was determined through active tracing, including contact with attending physicians, patient households, and consultation of the civil registry to ascertain vital status and date of death when applicable. Patients were censored at the date of the last confirmed follow-up.

Clinical and pathological assessment

The diagnosis of gastric adenocarcinoma was confirmed histologically via upper gastrointestinal endoscopic biopsies or surgical specimens. Preoperative staging was performed via contrast-enhanced computed tomography, supplemented by endoscopic ultrasonography when available. The tumor location was initially determined on the basis of endoscopic and radiological findings and subsequently confirmed intraoperatively. Pathological staging was based on the 2017 Union for International Cancer Control (UICC) TNM classification [16], with all patients restaged accordingly, to ensure uniformity across the study period. Histopathological evaluation included tumor size, depth of invasion, lymph node involvement, resection margin status, and presence of serosal invasion, as reported in the original pathology records.

Surgical procedures and operational definitions

Patients who underwent gastrectomy for gastric cancer were identified from institutional surgical records. Subtotal or total gastrectomy was performed according to tumor location and extent, following institutional surgical practice during the study period.

Surgical resections were classified according to the completeness of tumor removal. R0 resection was defined as complete tumor excision with histologically negative margins. R1 resection corresponded to microscopic residual tumor at the resection margin, whereas R2 resection indicated macroscopic residual disease. Surgical procedures were categorized as curative-intent when complete tumor resection with lymphadenectomy was attempted, and as palliative when performed to relieve symptoms in the presence of unresectable or metastatic disease.

Lymphadenectomy was performed with curative intent whenever feasible; however, the extent of nodal dissection varied during the study period according to evolving surgical standards and patient-related factors.

Postoperative morbidity and mortality were defined as any complication or death occurring within 30 days after surgery. Anastomotic or duodenal stump fistulas were diagnosed based on clinical findings (e.g., peritonitis, persistent fever, or external digestive leakage) and/or radiological or endoscopic confirmation, including events occurring after hospital discharge.

To ensure methodological homogeneity, survival and recurrence analyses were restricted to patients who underwent curative-intent surgery and achieved an R0 resection. Patients who underwent palliative procedures or non-curative resections (R1/R2) were excluded from these analyses. Overall survival was calculated from the date of surgery to the date of death or last follow-up. Disease recurrence was defined as the occurrence of locoregional relapse and/or distant metastasis confirmed during follow-up.

Outcome definitions

Overall survival (OS) was defined as the interval between the date of surgery and death from any cause or the last follow-up. Progression-free survival (PFS) was defined as the time from surgery to documented disease recurrence, progression, or death, whichever occurred first.

Locoregional recurrence was defined as radiological or histologically confirmed tumor relapse in the gastric bed or regional lymph nodes during follow-up among patients who underwent curative (R0) resection, whether it occurred alone or concurrently with distant metastasis. Distant recurrence was defined as hematogenous or peritoneal metastatic spread, with these patterns analyzed separately.

In patients who underwent palliative resection, the detection of an anastomotic mass or metastatic lymphadenopathy was considered disease progression rather than locoregional recurrence.

Statistical analysis

Statistical analyses were performed via IBM SPSS Statistics, version 20.0 (IBM Corp., Armonk, NY, USA). Continuous variables were assessed for normality via the Kolmogorov-Smirnov test. Normally distributed variables are expressed as the means ± standard deviations, whereas nonnormally distributed variables are reported as medians with interquartile ranges. Categorical variables are summarized as frequencies and percentages.

Survival curves were estimated via the Kaplan-Meier method and compared via the log-rank test. Univariate and multivariate Cox proportional hazards regression analyses were conducted to identify factors associated with survival and recurrence outcomes. All variables with a p-value < 0.20 in the univariate analysis were considered for inclusion in the multivariate Cox proportional hazards model. The results are reported as hazard ratios (HRs) with 95% confidence intervals (CIs). The proportional hazards assumption was assessed graphically, and all tests were two-sided, with a p-value <0.05 considered statistically significant.

## Results

Clinical presentation and diagnostic assessment

A total of 192 patients were included in this study. Table 1 presents the clinical and demographic characteristics of patients with GC.

**Table 1:** Baseline clinical and demographic characteristics of patients with gastric cancer ASA: American Society of Anesthesiologists physical status classification; GC: gastric cancer. Percentages are calculated based on the total sample (N = 192). Risk factors and comorbidities are not mutually exclusive.

Characteristics	N	%
Age (years)		
Mean ± SD	58.9 ± 13.72	—
> 70	52	27.1
40–70	127	66.1
< 40	13	6.8
Gender		
Male	127	66.1
Female	65	33.9
ASA score		
I	106	55.2
II	70	36.4
III	13	6.8
IV	3	1.6
Risk factors		
Helicobacter pylori infection	52	27.1
Biermer disease	1	0.5
First-degree family history of gastric cancer	6	3.1
Family history of Lynch syndrome	3	1.6
Medical comorbidities		
Hypertension	54	28.1
Diabetes mellitus	31	16.1
Total	192	100

Among these patients, the most frequent presenting symptoms were epigastric pain (91.6%), significant weight loss (87.0%; mean 12.3 ± 4.2 kg), and vomiting (59.4%). A small subset (1.6%) presented with hemorrhagic shock requiring emergency intervention. The preoperative characteristics are summarized in Table 2, showing that tumors were predominantly located in the lower (50.0%) and middle thirds (40.1%) of the stomach, with most patients presenting with uncomplicated forms (73.4%).

**Table 2:** Preoperative staging and radiologic findings in patients with gastric cancer Values are presented as N. Staging was based on preoperative imaging. * Multiple loco-regional findings may be present in the same patient. † Multiple sites of invasion and metastasis may coexist in the same patient.

Features	N	%
Tumor location		
Upper third	13	6.8
Middle third	77	40.1
Lower third	96	50.0
Gastric stump	6	3.1
Clinical form		
Uncomplicated forms	141	73.4
Complicated forms – stenosis	48	25.0
Complicated forms – hemorrhage	3	1.6
Loco-regional extension*		
Gastric wall thickening	156	81.2
Perigastric lymph nodes	80	41.7
Hepatic pedicle lymph nodes	30	15.6
Celiac lymph nodes	25	13.0
Interaortocaval lymph nodes	3	1.6
Adjacent organ invasion†		
Liver (left lobe)	2	1.0
Duodenum (D1)	3	1.6
Pancreas	3	1.6
Transverse colon	1	0.5
Spleen	1	0.5
Metastatic extension†		
Lung metastases	6	3.1
Liver metastases	6	3.1
Ovarian metastases	2	1.0
Peritoneal carcinomatosis	2	1.0

Upper gastrointestinal endoscopy, performed in all patients, identified antral tumors in 55.2%, which were classified as ulcero-vegetative (50.5%), ulcerated (33.3%), or infiltrative (11.5%). Linitis plastica was suspected in 25.7% of the patients. Preoperative imaging included abdominal computed tomography (73.4%), ultrasound (18.8%), magnetic resonance imaging (0.5%), and endoscopic ultrasound (1.6%), reflecting real-world resource availability. Loco-regional extension was frequent, particularly gastric wall thickening (81.2%), as detailed in Table 2.

Surgical procedures

Surgery was performed in 98.4% of the patients (n = 189), with a median delay from diagnosis to intervention of three days (IQR: 2-7). Emergency surgery was required in 1.6% of patients due to uncontrolled hemorrhage.

Total gastrectomy was performed in 49% and subtotal gastrectomy in 51% of the patients. Lymphadenectomy was D1 in 31.3%, D1.5 in 33.9%, and D2 in 34.9%, and multivisceral resection was performed in 17.2%, most frequently including splenectomy (9.4%).

The 30-day postoperative mortality rate was 7.3% (n = 14). Table 3 provides detailed patient-level data on these deaths. Mortality was predominantly attributable to medical causes, particularly sudden cardiac arrest and acute respiratory failure; whereas surgical causes were mainly related to intra-abdominal sepsis due to anastomotic or duodenal stump leaks, as well as postoperative hemorrhage.

**Table 3:** Etiologies and clinical characteristics of postoperative mortality (N = 14) ASA: American Society of Anesthesiologists physical status classification. Each row represents an individual patient; therefore, similar causes of death may occur in more than one patient.

Patient No.	Cause of death	Age (years)	ASA	Emergency surgery	Time to death (days)
Medical causes					
1	Altered mental status (encephalopathy)	76	III	No	3
2	Ischemic stroke	62	I	No	1
3	Sudden cardiac arrest	39	I	No	1
4	Pneumonia	47	II	Yes	12
5	Acute respiratory failure (non-septic)	57	II	Yes	30
6	Acute respiratory failure (non-septic)	65	II	No	8
7	Sudden cardiac arrest	74	III	No	12
8	Sudden cardiac arrest	81	III	No	5
Surgical causes					
9	Peritonitis due to duodenal stump leak	30	I	No	3
10	Peritonitis due to gastrojejunal anastomotic leak	72	II	No	8
11	Peritonitis due to esophagojejunal anastomotic leak	83	II	No	7
12	Peritonitis (unspecified source)	78	II	No	10
13	Postoperative hemorrhage	62	II	No	4
14	Postoperative hemorrhage	56	II	Yes	18

Overall, postoperative complications occurred in 13.5% of patients, including anastomotic leaks (2.1%), duodenal stump fistulas (4.2%), and intra-abdominal abscesses (6.3%), with reintervention required in a subset of cases. Medical complications were observed in 16.1% of patients, most commonly pneumonia (8.3%) and acute kidney injury (4.2%).

Pathological characteristics

The mean tumor size was 5.4 cm (range: 1.2-12 cm). According to Lauren’s classification, 53.2% were diffuse-type, frequently with signet-ring morphology. The tumors were poorly differentiated in 44.2%, moderately differentiated in 33.2%, and well differentiated in 7.4% of the patients. Linitis plastica was confirmed in 32.1% of the patients.

Among the curative-intent resections, R0 resection was achieved in 85.6% of the patients, with mean proximal and distal margins of 4.77 cm and 2.7 cm, respectively. The median number of lymph nodes retrieved was 13 (range: 0-52), with 42.6% of specimens yielding ≤10 nodes, reflecting challenges in adequate nodal harvest in a real-world setting.

Concordance of preoperative and pathological staging

Preoperative staging underestimates disease extent in several domains. Lymph node metastases were undetected in 26.6% of patients, whereas 17.2% were misclassified as node negative. Metastatic disease, including hepatic (9.4%), peritoneal (6.3%), and ovarian (1.6%) metastases, was identified intraoperatively in 17.2% of patients initially staged as M0.

Discordance was also noted for linitis plastica (false positives, 2.6%; false negatives, 7.3%) and Borrmann classification (discordant in 22.9%). The T stage was underestimated in 28.1% of patients, primarily due to unrecognized serosal (T4a, 19.8%) or adjacent organ invasion (T4b, 8.3%).

Oncologic treatment and survival outcomes

Perioperative chemotherapy was administered in nine patients, adjuvant chemotherapy in 12 patients, and adjuvant chemoradiotherapy in nine patients, reflecting historical treatment patterns.

The median overall survival (OS) was 13 months (95% CI: 10.2-15.8), with a 5-year OS of 22%. In multivariate Cox regression analysis, larger tumor size (aHR 1.13, 95% CI: 1.04-1.19, p = 0.002), advanced stage (aHR 1.63, 95% CI: 1.05-2.53, p = 0.027), and higher number of positive lymph nodes (aHR 1.13, 95% CI: 1.08-1.18, p < 0.001) were independently associated with worse overall survival. Non-curative resection was also associated with poor prognosis (aHR 3.21, 95% CI: 2.21-4.66, p < 0.001) (Table 4).

**Table 4:** Univariate and multivariate Cox regression analysis of factors associated with overall survival ASA: American Society of Anesthesiologists physical status classification; BMI: body mass index; CA 19-9: carbohydrate antigen 19-9; CEA: carcinoembryonic antigen; HR: hazard ratio; aHR: adjusted hazard ratio; CI: confidence interval. Variables included in the multivariate model were selected based on a univariate p-value < 0.20. A p-value < 0.05 was considered statistically significant.

Risk factor	Univariate HR	95% CI	p	Adjusted HR (aHR)	95% CI	p
Patient-related factors						
Gender	0.93	0.65–1.33	0.720	—	—	—
Age	1.13	0.81–1.57	0.445	—	—	—
BMI	0.88	0.36–2.16	0.780	—	—	—
ASA score	1.02	0.53–1.95	0.938	—	—	—
Tumor-related factors						
Tumor size	1.13	1.06–1.20	<0.001	1.13	1.04–1.19	0.002
Macroscopic type	0.97	0.76–1.23	0.059	0.84	0.63–1.13	0.269
Infiltrating component	1.57	1.09–2.28	0.013	1.15	0.69–1.91	0.573
Tumor location	0.79	0.51–1.22	0.061	0.79	0.56-1.12	0.190
Serosal invasion	2.38	1.63–3.46	<0.001	1.07	0.65–1.75	0.786
Number of positive lymph nodes	1.03	1.01–1.05	<0.001	1.13	1.08-1.18	<0.001
Lymph node ratio (LNR)	3.23	3.01–5.04	<0.001	1.55	0.87–2.75	0.135
Linitis plastica	1.40	0.98–1.99	0.053	1.19	0.77–1.84	0.430
Stage	2.89	2.05–4.04	<0.001	1.63	1.05–2.53	0.027
Intervention-related factors						
Emergency surgery	1.67	0.53–5.28	0.364	—	—	—
Type of gastrectomy	1.26	0.94–1.68	0.261	—	—	—
Lymphadenectomy	1.59	1.29–1.97	<0.001	1.16	0.89–1.50	0.250
Resection margin status	1.98	1.53–2.57	<0.001	2.18	1.57-3.03	<0.001
Curative resection	3.45	2.43–4.88	<0.001	3.21	2.21–4.66	<0.001
Postoperative complications	1.09	0.62–1.89	0.747	—	—	—
Treatment-related factors						
Perioperative chemotherapy	0.77	0.34–1.76	0.537	—	—	—
Adjuvant chemotherapy	1.08	0.63–1.86	0.756	—	—	—

Recurrence patterns

Among 116 patients who underwent curative-intent resection, the recurrence-free survival (RFS) rates at 1, 3, and 5 years were 83%, 68%, and 68%, respectively. Locoregional recurrence occurred in 21.7% of patients, most commonly involving nodal sites (n = 16), followed by anastomotic (n = 5) and combined sites (n = 2). The mean time to recurrence is 17 ± 3 months. 

In univariate analysis, the type of gastrectomy was significantly associated with locoregional recurrence (HR 2.81, 95% CI: 1.07-7.39, p = 0.027), while tumor location showed a trend toward association (p = 0.060). In multivariate analysis, subtotal gastrectomy remained independently associated with an increased risk of locoregional recurrence (aHR 2.81, 95% CI: 1.07-7.32, p = 0.034) (Table 5).

**Table 5:** Univariate and multivariate Cox regression analysis of factors associated with locoregional recurrence CEA: carcinoembryonic antigen; CA 19-9: carbohydrate antigen 19-9; HR: hazard ratio; aHR: adjusted hazard ratio; CI: confidence interval. Variables included in the multivariate model were selected based on a univariate p-value < 0.20. A p-value < 0.05 was considered statistically significant.

Risk factor	Univariate HR	95% CI	p	Adjusted HR (aHR)	95% CI	p
Patient-related factors						
Gender	0.81	0.33–2.01	0.656	—	—	—
Age (> 60 vs ≤ 60 years)	2.15	0.81–5.69	0.111	1.75	0.65–4.68	0.263
CA 19-9	1.00	0.99–1.00	0.993	—	—	—
CEA	0.99	0.97–1.01	0.662	—	—	—
Tumor-related factors						
Tumor size	1.00	0.77–1.29	0.982	—	—	—
Signet ring cell histology	0.67	0.28–1.16	0.379	—	—	—
Linitis plastica	1.05	0.30–3.67	0.928	—	—	—
Tumor location	0.52	0.23–1.17	0.060	1.70	0.29–9.77	0.551
Serosal invasion	1.26	0.36–4.37	0.706	—	—	—
Number of dissected lymph nodes	1.11	0.48–2.59	0.794	—	—	—
Number of positive lymph nodes	0.98	0.93–1.04	0.708	—	—	—
Lymph node ratio (LNR)	0.59	0.11–3.07	0.531	—	—	—
Tumor differentiation grade	0.80	0.51–1.26	0.343	—	—	—
T stage	0.89	0.46–1.72	0.737	—	—	—
Vascular invasion	0.85	0.35–2.08	0.734	—	—	—
Perineural invasion	0.96	0.38–2.39	0.936	—	—	—
Intervention-related factors						
Type of gastrectomy	2.81	1.07–7.39	0.027	2.81	1.07–7.32	0.034
Postoperative complications	3.65	1.96–16.08	0.061	2.72	1.69–10.63	0.208
Treatment-related factors						
Adjuvant chemotherapy	1.31	0.29–5.77	0.716	—	—	—

Factors associated with recurrence

Distant metastases were observed in 41 patients. The most frequent patterns were peritoneal carcinomatosis (25.9%) and hematogenous spread (15.5%). The median time to distant recurrence was 21 months. Patients presenting with multiple metastatic sites were grouped and analyzed as a single distant recurrence category.

In univariate analysis, macroscopic tumor features (HR 1.70, 95% CI: 1.19-2.42, p = 0.007) and infiltrative tumor growth (HR 2.86, 95% CI: 1.36-6.01, p = 0.003) were significantly associated with distant recurrence. In multivariate analysis, infiltrative tumor growth (aHR 3.49, 95% CI: 1.59-7.63, p = 0.002) and serosal invasion (aHR 2.88, 95% CI: 1.16-7.18, p = 0.023) remained independent predictors, while other variables were not significantly associated (Table 6).

**Table 6:** Univariate and multivariate Cox regression analysis of factors associated with distant recurrence HR, hazard ratio; aHR, adjusted hazard ratio; CI, confidence interval; BMI, body mass index; LNR, lymph node ratio; CEA, carcinoembryonic antigen; CA 19-9, carbohydrate antigen 19-9. Variables included in the multivariate model were selected based on a univariate p-value < 0.20. A p-value < 0.05 was considered statistically significant.

Variables	Univariate HR	95% CI	p-value	Adjusted HR (aHR)	95% CI	p-value
Patient-related factors						
Gender	0.97	0.51–1.84	0.933	—	—	—
Age	1.74	0.89–3.41	0.094	1.14	0.42–3.11	0.789
BMI	0.99	0.90–1.09	0.945	—	—	—
Tumor-related factors						
Tumor size	0.99	0.87–1.13	0.937	—	—	—
Macroscopic features	1.70	1.19–2.42	0.007	0.72	0.30–1.69	0.454
Infiltrative growth	2.86	1.36–6.01	0.003	3.49	1.59–7.63	0.002
Tumor location	0.99	0.23–4.18	0.992	—	—	—
Serosal invasion	1.87	0.80–4.36	0.127	2.88	1.16–7.18	0.023
Number of positive lymph nodes	0.99	0.95–1.03	0.682	—	—	—
Lymph node ratio (LNR)	0.74	0.28–1.93	0.546	—	—	—
Linitis plastica	1.87	0.88–3.96	0.089	1.61	0.72–3.55	0.238
T stage	1.28	0.74–2.22	0.310	—	—	—
Overall stage	0.84	0.45–1.59	0.595	—	—	—
Tumor differentiation	0.90	0.61–1.32	0.848	—	—	—
Vascular invasion	0.99	0.53–1.86	0.986	—	—	—
Perineural invasion	0.90	0.48–1.69	0.775	—	—	—
CA 19-9	1.00	0.99–1.01	0.080	1.00	0.99–1.01	0.485
Intervention-related factors						
Type of gastrectomy	1.24	0.66–2.32	0.483	—	—	—
Lymphadenectomy	0.87	0.57–1.35	0.553	—	—	—
Treatment-related factors						
Perioperative chemotherapy	1.66	0.57–4.83	0.336	—	—	—
Adjuvant chemotherapy	2.27	0.76–6.77	0.125	2.28	0.74–6.97	0.148
Postoperative chemoradiotherapy	1.75	0.41–7.52	0.436	—	—	—

## Discussion

GC remains a major global health burden, with survival outcomes still largely driven by late presentation and disparities in access to multidisciplinary treatment. In this context, our study provides valuable real-world data from a 16-year cohort of 192 consecutive, unselected patients treated in a tertiary referral center. The relative homogeneity of the surgical approach, which is largely based on open laparotomy performed by a consistent team of six surgeons, offers a clearer understanding of outcomes achievable under standardized yet resource-constrained conditions.

The median age of our cohort (58.9 ± 13.72 years) places Tunisia in an intermediate epidemiological zone, older than cohorts from sub-Saharan Africa (e.g., Nigeria: 48 years) but younger than those in high-income countries (e.g., Lebanon: 72 years; Spain: 67.9 years) [1,3,8]. This age gradient likely reflects regional variations in risk factor distribution, including a high prevalence of *H. pylori *(70% in Tunisia vs. 30% in Western countries) and dietary habits (e.g., high salt-preserved food, low fruit intake) [1,3,9]. Tunisia’s relatively younger demographic profile compared with Europe may also skew the age at presentation despite similar environmental exposures.

The observed male predominance (sex ratio: 1.95) is consistent with global data (male: female incidence ≈ 1.8:1) and may be attributed to both biological and behavioral factors. Estrogen-mediated upregulation of TFF1 has been proposed as a protective mechanism in females,10 whereas increased tobacco use (42% in Tunisian men vs. 2% in women)11 and occupational exposure (e.g., to nitrosamines) are likely contributors to increased male susceptibility [15].

Although 28.1% of patients had comorbidities (notably hypertension: 16.1%; diabetes: 8.3%), these comorbidities were not associated with survival outcomes, which contrasts with studies linking diabetes to poorer GC prognosis through hyperinsulinemia-driven tumor proliferation [17]. Possible explanations include selection bias (patients with severe comorbidities may have been deemed unfit for surgery) and the dominance of aggressive tumor biology in our cohort, specifically, the high proportion of signet ring cell carcinomas (38.3% vs. 14% in India), which may overshadow the influence of comorbid conditions on survival [18].

The elevated prevalence of signet ring cell carcinoma in our series, which surpassed that reported in Western studies (11-14%) but was comparable to that reported for metastatic GC (32.1%) [18], may reflect underlying molecular and environmental factors. Potential mechanisms include enrichment of CDH1 mutations (associated with hereditary diffuse-type GC) and CLDN18-ARHGAP fusions, both of which are under investigation as therapeutic targets [19]. Additionally, the synergistic effects of *H. pylori* virulence factors (e.g., CagA) and high-salt diets may promote epithelial-mesenchymal transition (EMT) and diffuse-type histology [20].

The predominance of stage III disease (62% of cases) is comparable to patterns reported in Arab countries [4,21]. Delayed presentation in our setting is likely multifactorial: early GC symptoms (e.g., dyspepsia, bloating) are nonspecific and frequently misattributed, and endoscopic access remains limited.

To address these trends, we propose a risk-stratified screening approach that targets high-risk individuals (e.g., males >50 years, *H. pylori*-positive, family history of GC) via noninvasive biomarkers (e.g., Gastropanel®) followed by endoscopy. Establishing a national molecular registry capturing HER2, Epstein-Barr virus (EBV), and microsatellite instability (MSI) status would facilitate patient stratification for targeted and immunotherapies [22]. Parallel efforts should focus on dietary interventions, such as national campaigns to reduce salt consumption and promote fruit and vegetable intake, such as successful models in Japan [4].

Although multimodal treatment (surgery and chemotherapy) is standard for locally advanced GC, adjuvant therapy utilization was suboptimal in our cohort, reflecting the absence of standardized treatment protocols during the study period. This highlights the need for evidence-based regional guidelines tailored to available resources.

The median overall survival (OS) of 13 months in our series reflects the aggressive nature of GC in Tunisia and aligns with regional data (Egypt: 11 months; Iran: 16.3 months; Turkey: 18 months) [1]. This narrow survival range across low- and middle-income countries (LMICs) underscores ongoing challenges related to late-stage diagnosis and limited access to multimodal care, contrasting sharply with high-income settings where the median OS exceeds 24 months in trials involving perioperative chemotherapy [1,4].

Multivariate Cox regression identified three factors associated with poor OS. First, tumor size (HR = 1.13, p = 0.002) with each 1 cm increase in tumor diameter was associated with a 13% higher mortality risk, which is consistent with the literature linking tumor volume to increased vascular invasion, genomic instability, and metastatic potential [4,23]. Second, patients with advanced-stage disease (HR = 1.63, p = 0.027) and patients with stage III/IV disease had a 63% greater risk of death, reinforcing the prognostic validity of the TNM staging system [24]. Notably, 85% of our cohort presented at an advanced stage, underscoring the need for public awareness campaigns to reduce diagnostic delays. Finally, R0 resection (HR = 3.21, p<0.001) with noncurative resection (R1/R2) was associated with a 3.21-fold increased risk of death, echoing meta-analyses showing a 10-15% survival advantage with complete (R0) resection [24].

Although signet ring histology was not an independent prognostic factor (p = 0.12), the observed trend toward reduced median OS (10 months vs. 15 months in nonsignet cases) aligns with meta-analyses reporting 20-30% lower five-year survival in this histological subtype [25].

Recurrence occurred in 58% of patients at a median interval of 9.2 months. Risk factors for distant recurrence include serosal invasion (HR = 2.88, p = 0.023), with tumors penetrating the serosa (pT4) significantly increasing the risk of peritoneal metastases, which is consistent with the "seed and soil" theory of free cancer cell dissemination [26], and an infiltrative growth pattern (HR = 3.49, p = 0.002) with diffuse-type tumors, characteristic of EMT activation and poor cellular cohesion, which are associated with increased recurrence risk [27].

For locoregional recurrence, subtotal gastrectomy (HR = 2.81, p = 0.008) was associated with a significantly greater hazard than total gastrectomy was, potentially because of residual nodal basins (e.g., undissected station 10 nodes) [28]. Our recurrence rate (34%) exceeded that reported in Japanese series employing D2 dissections (15-20%) [29], suggesting the need to adopt more comprehensive nodal clearance. Our peritoneal recurrence rate (42%) lies between Western (30%) and Eastern (55%) benchmarks [30], potentially reflecting intermediate lymphadenectomy (D1.5) and ethnobiological factors such as EBV-associated GC incidence [31].

This study underscores critical opportunities for improving GC outcomes through earlier detection, standardized surgical management, and broader access to adjuvant therapies. Screening high-risk populations via noninvasive biomarkers could facilitate earlier-stage diagnoses, whereas surgical centralization (≥20 gastrectomies/year/surgeon) may improve R0 resection rates and reduce postoperative complications. Expanding access to chemotherapy and radiotherapy (particularly in underserved regions) will require policy-level investment and infrastructure development. Integrating molecular profiling (e.g., HER2, MSI, and CLDN18.2) into routine practice can enable personalized treatment strategies, advancing precision oncology [23]. Collectively, these efforts demand coordinated action from clinicians, public health systems, and policymakers to reduce disparities and standardize high-quality care for patients with gastric cancer worldwide.

The findings of this study have several important clinical and policy implications. The predominance of late-stage diagnoses underscores the urgent need for national awareness campaigns and early detection strategies tailored to Tunisian epidemiology. Implementing risk-based screening protocols, expanding access to endoscopy, and integrating noninvasive biomarkers could substantially shift diagnosis toward earlier stages.

In surgical practice, the demonstrated prognostic value of R0 resection supports the centralization of gastrectomy procedures to high-volume centers capable of providing comprehensive lymphadenectomy and postoperative monitoring. Additionally, the suboptimal use of adjuvant therapies highlights the necessity of establishing clear, evidence-based national treatment guidelines aligned with international standards but adapted to local resources. Integration of molecular testing for HER2, MSI, and emerging biomarkers such as CLDN18.2 may further enable access to targeted and immunotherapies, positioning precision oncology as a feasible goal in the Tunisian context.

Study limitations

Several limitations must be acknowledged to contextualize the study’s findings. Its retrospective, single-center design inherently introduces selection bias and limits generalizability. Evolving diagnostic and treatment strategies over 16 years may introduce variability in staging and outcomes. Underreporting of minor postoperative complications, particularly minor or conservatively managed events, may underestimate morbidity rates. Additionally, limited molecular data and inconsistent use of adjuvant therapy restrict the exploration of genetic determinants and treatment effects. Finally, the limited availability and inconsistent use of adjuvant therapy may influence survival and recurrence patterns, and these findings should be interpreted in the context of resource-limited healthcare settings.

## Conclusions

This study presents the first Tunisian assessment of treatment outcomes in patients with gastric cancer managed at a tertiary care center. In this cohort of 192 patients followed over 16 years, gastric cancer was predominantly diagnosed at advanced stages and was associated with poor prognosis, reflected by a median overall survival of 13 months and frequent recurrence.

Although surgery remains the cornerstone of curative management, our findings suggest an association between the use of perioperative and adjuvant chemotherapy and improved survival, supporting the relevance of a multimodal therapeutic approach in this setting. These observations highlight the need to strengthen early detection strategies, increase public awareness, and promote the implementation of standardized national management guidelines adapted to the Tunisian context. Strengthening multicenter collaborations across North Africa will also be important for generating robust regional evidence and improving gastric cancer care.

## References

[1] Morgan E, Arnold M, Camargo MC (2022). The current and future incidence and mortality of gastric cancer in 185 countries, 2020-40: A population-based modelling study. EClinicalMedicine.

[2] Bray F, Laversanne M, Sung H, Ferlay J, Siegel RL, Soerjomataram I, Jemal A (2024). Global cancer statistics 2022: GLOBOCAN estimates of incidence and mortality worldwide for 36 cancers in 185 countries. CA Cancer J Clin.

[3] Ajani JA, D'Amico TA, Almhanna K (2016). Gastric cancer, version 3.2016, NCCN clinical practice guidelines in oncology. J Natl Compr Canc Netw.

[4] Sundar R, Nakayama I, Markar SR, Shitara K, van Laarhoven HW, Janjigian YY, Smyth EC (2025). Gastric cancer. Lancet.

[5] El Halabi M, Horanieh R, Tamim H (2020). The impact of age on prognosis in patients with gastric cancer: experience in a tertiary care centre. J Gastrointest Oncol.

[6] Díaz Del Arco C, Ortega Medina L, Estrada Muñoz L, Molina Roldán E, García Gómez de Las Heras S, Fernández Aceñero MJ (2023). Impact of age at diagnosis on clinicopathological features, prognosis, and management of gastric cancer: a retrospective single-center experience from Spain. Cancers (Basel).

[7] Bilici A, Selcukbiricik F (2015). Prognostic significance of the recurrence pattern and risk factors for recurrence in patients with proximal gastric cancer who underwent curative gastrectomy. Tumour Biol.

[8] Charles L, Elangovan A, Nisha Y (2024). Clinicopathological features and survival outcomes for gastric adenocarcinoma: real-world single-center data. Indian J Gastroenterol.

[9] Morishima T, Matsumoto Y, Koeda N (2019). Impact of comorbidities on survival in gastric, colorectal, and lung cancer patients. J Epidemiol.

[10] (2014). Comprehensive molecular characterization of gastric adenocarcinoma. Nature.

[11] Ramaswamy A, Bhargava P, Ostwal V (2020). Real-world long-term outcomes with perioperative EOX in D2 gastrectomy: a meaningful look while we switch to FLOT-4. J Gastrointest Cancer.

[12] Busweiler LA, Henneman D, Dikken JL (2017). Failure-to-rescue in patients undergoing surgery for esophageal or gastric cancer. Eur J Surg Oncol.

[13] Bang YJ, Kim YW, Yang HK (2012). Adjuvant capecitabine and oxaliplatin for gastric cancer after D2 gastrectomy (CLASSIC): a phase 3 open-label, randomised controlled trial. Lancet.

[14] Lee J, Lim DH, Kim S (2012). Phase III trial comparing capecitabine plus cisplatin versus capecitabine plus cisplatin with concurrent capecitabine radiotherapy in completely resected gastric cancer with D2 lymph node dissection: the ARTIST trial. J Clin Oncol.

[15] Wu J, Tian S, Xu J, Cheng N, Chen X, Yin J, Nie Z (2023). Association of high-risk comorbidity with overall survival among patients with gastric cancer and its sex-specific differences in China: a retrospective observational cohort study. BMC Cancer.

[16] Brierley JD, Gospodarowicz MK, Wittekind C (2017). The TNM classification of malignant tumours, 8th edition. The TNM classification of malignant tumours, 8th edition.

[17] Baiocchi GL, Giacopuzzi S, Reim D (2020). Incidence and grading of complications after gastrectomy for cancer using the GASTRODATA registry: a European retrospective observational study. Ann Surg.

[18] Basaran H, Koca T, Cerkesli AK, Arslan D, Karaca S (2015). Treatment outcomes and survival study of gastric cancer patients: a retrospective analysis in an endemic region. Asian Pac J Cancer Prev.

[19] Toriumi T, Terashima M, Mizusawa J (2023). Recurrence patterns after curative gastrectomy for pStage II/III gastric cancer: Exploratory analysis of the randomized controlled JCOG1001 trial. Eur J Surg Oncol.

[20] Jiao X, Wang Y, Wang F, Wang X (2020). Recurrence pattern and its predictors for advanced gastric cancer after total gastrectomy. Medicine (Baltimore).

[21] Lv L, Liang X, Wu D (2021). Is cardia cancer a special type of gastric cancer? A differential analysis of early cardia cancer and non-cardia cancer. J Cancer.

[22] Ajani JA, D'Amico TA, Bentrem DJ (2022). Gastric cancer, version 2.2022, NCCN clinical practice guidelines in oncology. J Natl Compr Canc Netw.

[23] Wagner AD, Syn NL, Moehler M (2017). Chemotherapy for advanced gastric cancer. Cochrane Database Syst Rev.

[24] Karimi P, Islami F, Anandasabapathy S, Freedman ND, Kamangar F (2014). Gastric cancer: descriptive epidemiology, risk factors, screening, and prevention. Cancer Epidemiol Biomarkers Prev.

[25] Sano T, Coit DG, Kim HH (2017). Proposal of a new stage grouping of gastric cancer for TNM classification: International Gastric Cancer Association staging project. Gastric Cancer.

[26] Thrift AP, El-Serag HB (2020). Burden of gastric cancer. Clin Gastroenterol Hepatol.

[27] Anderson WF, Rabkin CS, Turner N, Fraumeni JF Jr, Rosenberg PS, Camargo MC (2018). The changing face of Noncardia gastric cancer incidence among US non-Hispanic whites. J Natl Cancer Inst.

[28] Sasako M, Sakuramoto S, Katai H (2011). Five-year outcomes of a randomized phase III trial comparing adjuvant chemotherapy with S-1 versus surgery alone in stage II or III gastric cancer. J Clin Oncol.

[29] Cunningham D, Allum WH, Stenning SP (2006). Perioperative chemotherapy versus surgery alone for resectable gastroesophageal cancer. N Engl J Med.

[30] Songun I, Putter H, Meershoek-Klein Kranenbarg E, Sasako M, van de Velde CJ (2010). Surgical treatment of gastric cancer: 15-year follow-up results of the randomised nationwide Dutch D1D2 trial. Lancet Oncol.

[31] Degiuli M, De Manzoni G, Di Leo A (2016). Gastric cancer: current status of lymph node dissection. World J Gastroenterol.

